# Efficient Large‐Area Graphene *p‐n* Junction Terahertz Receivers on an Integrated Optical Platform

**DOI:** 10.1002/smtd.202500083

**Published:** 2025-04-17

**Authors:** Leonardo Viti, Vladimir Pushkarev, Syed Muhammad Abouzar Sarfraz, Gaetano Scamarcio, Miriam S. Vitiello

**Affiliations:** ^1^ NEST CNR‐Istituto Nanoscienze and Scuola Normale Superiore Piazza San Silvestro 12 Pisa 56127 Italy; ^2^ Dipartimento Interateneo di Fisica Università degli studi di Bari Via Amendola 173 Bari Italy

**Keywords:** graphene, Salisbury screen, terahertz

## Abstract

The integration of graphene‐based *p‐n* junctions into photonic or optoelectronic platforms can allow efficient guiding and absorption of the light signals into the detection element, promising a major improvement in the efficiency of the system, by minimizing optical losses and enhancing the light coupling. These platforms can also potentially provide additional functionalities, such as frequency filtering, modulation, or multiplexing of the signals. At terahertz (THz) frequencies, this can lead to a variety of new applications such as sensing, imaging, and communication, as well as advancements in high‐speed electronics and wireless technologies. Here, we took advantage of large area industrial‐scale graphene, realized via an inexpensive production process, to engineer an antenna‐integrated graphene Salisbury screen (AgSS) *p‐n* junction photodetector, in which the electromagnetic coupling between graphene and the free‐space wavelength is optimized by controlling the antenna dimensions and its distance from a sub‐wavelength thin reflective mirror. Room‐temperature noise equivalent powers < 300 pWHz^−1/2^, response time < 5 ns and a power dynamic range larger than four orders of magnitude at 2.86 THz is reached, exceeding the performances of exfoliated graphene photodetectors technologies, and competitive with complementary metal‐oxide semiconductor (CMOS) and micro‐bolometer technologies at high THz frequencies.

## Graphical abstract

An antenna‐integrated graphene Salisbury screen (AgSS) *p‐n* junction is engineered to detect radiation at 2.8 THz with room‐temperature noise equivalent powers < 300 pWHz^−1/2^, response time < 5 ns and a power dynamic range larger than four orders of magnitude.

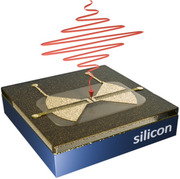

## 1 Introduction

Integrated optoelectronic systems and networks, nowadays require fast (tens of GHz bandwidth) sensitive (noise equivalent powers of a few pW/Hz^1/2^), and broad dynamic range receivers to meet the demand for high data transmission rates (terabits per sec).^[^ ^1^, ^2^ ^]^ Recent advancements in miniaturized platforms for device integration, combined with the growing demand for parallelization – particularly in emerging fields such as energy harvesting,^[^ ^3^ ^]^ telecommunications,^[^ ^4^ ^]^ quantum technologies,^[^ ^5^ ^]^ robotics^[^ ^6^ ^]^ and imaging^[^ ^7^ ^]^ – have triggered major efforts in the development of novel large‐scale material platforms that are affordable, flexible, and easy to integrate with silicon‐based technologies. The developments of technologies based on single‐layer graphene (SLG), and other two‐dimensional (2D) materials, had a remarkable expansion in recent years.^[^ ^8^, ^9^, ^10^, ^11^ ^]^ As a notable example, graphene has led to a breakthrough in the field of photodetectors, due to its high charge carrier mobility, broadband absorption, low footprint, and ability to operate across a wide range of wavelengths, including ultraviolet (UV),^[^ ^12^ ^]^ visible,^[^ ^13^, ^14^ ^]^ near‐infrared (NIR, telecom),^[^ ^15^, ^16^, ^17^ ^]^ mid‐infrared (mid‐IR),^[^ ^18^ ^]^ terahertz (THz),^[^ ^19^, ^20^ ^]^ and sub‐THz.^[^ ^21^ ^]^ The possibility to adopt scalable synthesis methods has further facilitated the transition of graphene technologies from laboratory settings to a multi‐million‐dollar industrial and technological market,^[^ ^22^, ^23^ ^]^ with a forecast beyond 1 billion before 2030.^[^ ^24^ ^]^ In addition to traditional light detection systems, graphene‐based devices can also be used for light modulation and integratedn with other materials for enhanced performance, such as graphene–quantum dot^[^ ^15^ ^]^ or graphene–semiconductor hybrid devices.^[^ ^25^ ^]^

Micro‐ and nano‐photonics systems, with their unprecedented design degrees of freedom, have recently been leveraged to maximize the coupling of the 2D, atomically thin film with the optical field, thereby improving absorption in graphene. This has led to the successful combination of graphene with metamaterials,^[^ ^26^ ^]^ photonic integrated circuit (PIC) platforms,^[^ ^27^ ^]^ or frequency resonant elements, such as waveguides^[^ ^28^ ^]^ and antennas.^[^ ^18^ ^]^ These latter have primarily been employed at long wavelengths, specifically from the mid‐IR to the sub‐THz frequency ranges,^[^ ^18^, ^19^, ^21^ ^]^ owing to their ability to efficiently funnel the optical field into volumes much smaller than the diffraction limit, resulting in field enhancement factors of ≈100.^[^ ^19^ ^]^

Specifically, engineering resonator design and integrating specific antenna architectures on‐chip can allow to selectively tailor the mechanism of photodetection, controlling the specific areas of graphene where photo‐excitation occurs.^[^ ^20^, ^29^ ^]^ This functionality, combined with the possibility to tune the carrier density, the chemical potential, and the absorption, in graphene,  through external electrostatic gating, led to the broad developments of antenna‐coupled *p‐n* junction graphene photodetectors based on the hot‐carrier‐assisted photo‐thermoelectric (PTE) effect,^[^ ^18^, ^30^ ^]^ operating over a broad frequency range, spanning from the mid‐IR,^[^ ^31^ ^]^ to the sub‐THz domains.^[^ ^21^ ^]^ In these systems, infrared light, collected by the antenna, is absorbed in graphene, creating a temperature gradient (Δ*T*_e_, where *T*_e_ is the electronic temperature) in the electronic population. When this gradient spatially overlaps with a sharp inhomogeneity in the material, as in the case of an electrostatically defined *p‐n* junction, an electron‐heat‐driven photoresponse can be generated through the Seebeck effect. The photo thermoelectric voltage (*V*_PTE_) is then given by the product of the gradient of the Seebeck coefficient across the junction (Δ*S*_b_ = *S*_b_(left) – *S*_b_(right)) and the electronic temperature gradient: *V*_PTE_ = Δ*T*_e_ × Δ*S*_b_.

SLG can simultaneously host large *T*_e_ and *S*_b_ gradients. Indeed, light‐induced Δ*T*_e_ levels up to ≈1000 K can be achieved under illumination with NIR^[^ ^32^ ^]^ or THz^[^ ^33^ ^]^ photons. Strikingly, thanks to the small electronic heat capacitance of the electronic subsystem (≈2000 *k*_B_ µm^−2^ at room temperature,^[^ ^34^ ^]^ where *k*_B_ is the Boltzmann constant), and to the difference between the timescales for electron‐electron thermalization (≈100 fs)^[^ ^35^ ^]^ and electron cooling pathways (≈1–10 ps),^[^ ^36^, ^37^ ^]^ this can occur both for interband and intraband (Drude‐like) absorption. *S*_b_ values >150 µV K^−1^^[^ ^38^ ^]^ and ≈100 µV K^−1[^ ^16^ ^]^ can be obtained for high‐quality hBN‐encapsulated graphene and for large‐area graphene films synthesized by chemical vapor deposition (CVD), respectively. These features, combined with the ease of transfer and technological maturity of SLG,^[^ ^8^, ^10^ ^]^ make graphene an optimal candidate, among other 2D materials, for the development of PTE‐based far‐ and mid‐infrared photodetectors, especially when integrated onto existing optical platforms. Importantly, in SLG, *S*_b_ is a function of the carrier density and typically decreases when the material quality is reduced. We note that the *p‐n* junction geometry is ideal to take full advantage of the PTE conversion: the transition between *n*‐type doping and *p*‐type doping regions across the junction maximizes the gradient Δ*S*_b_. In addition, efficient THz graphene PTE detectors require a large field enhancement, localized at the junction position, as demonstrated in high‐quality graphene‐hBN layered material heterostructures (LMH),^[^ ^30^, ^39^ ^]^ and large‐area CVD‐grown graphene fabricated on various solid‐state‐based dielectrics.^[^ ^19^, ^40^ ^]^

Here, we conceive an alternative geometry that relies on the integration of *p‐n* junction photodetectors onto pre‐defined photonic platforms. Specifically, we embed a SLG, antenna‐coupled, field‐effect transistor on a THz Salisbury screen architecture, a device platform that recently enabled the development of efficient THz modulators,^[^ ^41^ ^]^ saturable absorbers,^[^ ^42^ ^]^ and communication components adopted, for example, in radiation protection and shielding.^[^ ^43^ ^]^

## 2 Results and Discussion

### 2.1 Device configuration

**Figure 1:**
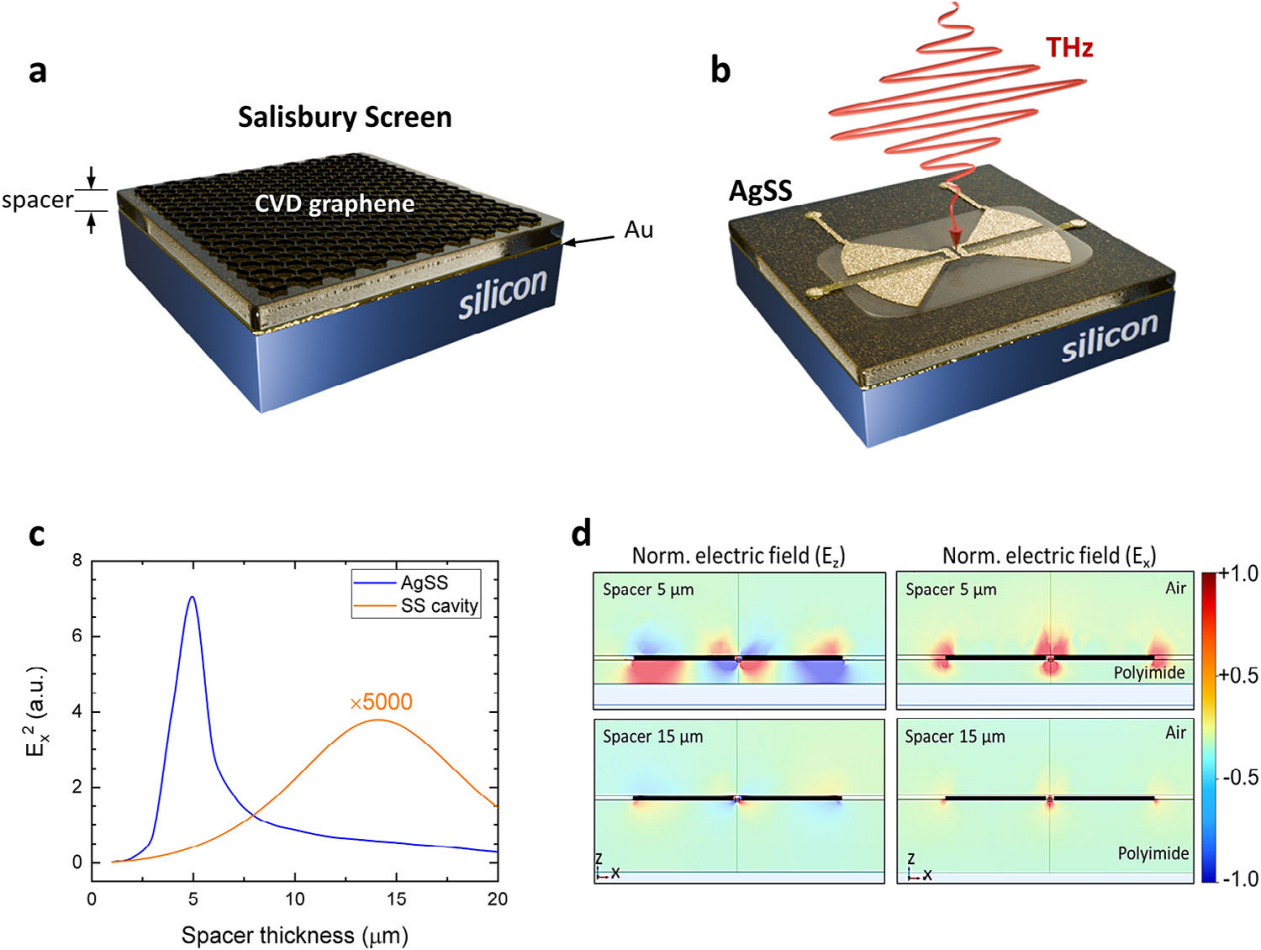
a) Schematic diagram of the SLG‐based THz coupler. The polyimide (PI) spacer thickness can be tuned by spin‐coating. A thick (200 nm) Au layer on silicon constitutes the substrate. b) Schematic view of the antenna‐coupled graphene Salisbury screen (AgSS) architecture. c) Finite element method simulations of the antenna‐coupled architecture. In the simulation tool, the 42 µm long antenna is excited with a plane wave, linearly polarized parallel to the dipole antenna axis. The electric field is then probed by averaging over a small volume placed at the center of the antenna gap (200 nm wide). The spacer thickness (*h*) strongly influences the antenna efficiency. A maximum field enhancement is visible for d = 5 µm. The comparison with a conventional Salisbury‐screen cavity (result multiplied by 5000 to improve visibility) shows orders of magnitude enhancement for the antenna‐coupled structure. d) Corresponding electric field distribution in proximity of the metallic antenna (cross‐sectional view, parallel to the plane of incidence). The left panels display the out‐of‐plane component of the electric field (*E*_z_). Right panels display the in‐plane component of the electric field (*E*_x_).

The device concept is schematically represented in **Figure** **1**a. In a graphene Salisbury screen (gSS), the graphene layer (absorber) is positioned at a quarter‐wavelength distance above a perfectly reflective surface. This configuration is achieved by adjusting the thickness (*h*) of a dielectric spacer layer. We integrate on‐chip a planar nano‐antenna on the gSS (Figure 1b). This design strategy is motivated by two key factors. First, the antenna‐coupled gSS system (AgSS) can provide a larger field enhancement (Figure 1c) compared to both the standalone antenna (>30 times larger) and the Salisbury screen cavity alone (>1000 times larger).

Additionally, the device architecture is substrate‐independent, hence facilitating dense co‐integration with rigid or flexible electronics. By leveraging this detector design and simultaneously optimizing the antenna shape, dimensions, and spacer layer thickness, we demonstrate AgSS *p‐n* junction PTE detectors with a noise equivalent power (NEP) <300 pWHz^−1/2^, response times *τ* ≈5 ns (setup‐limited), and a detection dynamic range exceeding four orders of magnitude at room temperature (RT), at 2.86 THz. These figures of merit surpass the previously reported performances of CVD‐based architectures in the same frequency range, bridging the gap between the sensitivity levels so far obtained with large‐area graphene (NEP ≈1 nWHz^−1/2^) and those obtained with high‐quality LMHs obtained by micromechanical cleavage of individual flakes (NEP ≈0.1 nWHz^−1/2^). When compared to the state‐of‐the‐art commercially available devices operating at frequencies ≈3 THz, the proposed technology is competitive with other existing platforms, such as silicon‐based complementary metal‐oxide‐semiconductor (CMOS) sensors, with NEP ≈100 pWHz^−1/2^ at 2.5 THz and *τ *≈1 ns,^[^ ^44^ ^]^ and cryogenically cooled microbolometers, which exhibit NEP ≈20 pWHz^−1/2^ and *τ* ≈10–1000 µs,^[^ ^45^ ^]^ but is simultaneously miniaturized, room temperature, cheap and suitable for multi‐pixel integration.

Despite the simplicity of the receiver concept, the realization of an efficient AgSS *p‐n* junction or THz detection requires an accurate selection and engineering of the geometrical and material parameters. The first fundamental aspect of the gSS geometry is the choice of the material for the spacer layer. Because of the long free‐space radiation wavelength (≈100 µm), a target *h* in the range of 5 to 20 µm, depending on the dielectric spacer's relative permittivity (ε_r_), has to be employed. This requirement imposes significant constraints on the available materials. On one side, thin films of polymers are typically unstable when used in the fabrication of small‐sized (sub‐µm) elements and good uniformity is difficult to achieve over large (>1 × 1 cm^2^) areas.^[^ ^46^ ^]^ On the other hand, the growth of oxide layers with a thickness ≈10 µm can be time‐consuming and difficult to up‐scale. Here, we employed solidified polyimide (PI, or Kapton), allowing the thickness to be optimized by adjusting the rotation speed with which the liquid‐phase solution is spun to coat the sample (see Experimental Section). This choice is motivated by multiple reasons. First, a wide range of thicknesses can be targeted by spin‐coating, from <1 µm to >20 µm,^[^ ^47^ ^]^ with a surface roughness ≈ 1 nm. Furthermore, PI shows good mechanical, chemical, and thermal robustness against the fabrication process of graphene field‐effect transistors, which includes plasma etching, chemical removal of polymers in acetone (50 °C), methyl isobutyl ketone (MIBK, 4‐methylpentan‐2‐one), and isopropanol, and thermal treatments up to 130 °C, e.g. during atomic layer deposition. As a final remark, the choice of a PI‐based spacer prospects the extension of the present fabrication approach to flexible device platforms, particularly appealing in wearable infrared optoelectronics.^[^ ^48^, ^49^ ^]^

The geometrical parameters of the AgSS system, i.e., the dimensions of the THz dipole antenna and the spacer thickness *h*, are defined by electromagnetic simulations. We employ commercial software (COMSOL Multiphysics),^[^ ^50^ ^]^ based on a finite element method (FEM), to numerically evaluate the device geometry that maximizes the field enhancement at the graphene *p‐n* junction. In the 3D model, the detector is illuminated under normal incidence by a 2.86 THz plane wave, polarized parallel to the (bow‐tie) antenna axis. We vary the antenna radius (*r*_A_) and *h* and monitor the enhancement of the in‐plane component of the electric field,2. (*E*_x_) as a function of these geometrical parameters. The comparison of the simulated field enhancement for an AgSS system with that of a bare Salisbury screen cavity (Figure 1c), shows that for an ideal combination of dimensions, *r*_A_ = 24 µm and *h* = 5 µm, the AgSS geometry provides > three orders of magnitude larger field enhancement values. Interestingly, the optimal value of *h* is ≈3 times smaller than the quarter‐wavelength distance for a radiation of 2.86 THz propagating in a PI film, whose refractive index in the THz range is *n*_PI_ = 1.85.^[^ ^51^ ^]^ This effect can be understood by inspecting the simulated electric field distribution in proximity of the antenna. When *h* is set to the quarter wavelength distance, the antenna near‐field does not couple with the reflecting surface underneath the spacer layer. Conversely, when *h* is reduced to ≈5 µm, the electromagnetic field is squeezed in the spacer thickness between the antenna and the metallic plane, which together constitute a more efficient coupling element with respect to both a standalone antenna or a standalone Salisbury screen cavity. The resulting field enhancement involves both the in‐plane (*E*_x_) and out‐of‐plane (*E*_z_) components, as demonstrated by the snapshots of electric field distribution presented in Figure 1d.

**Figure 2:**
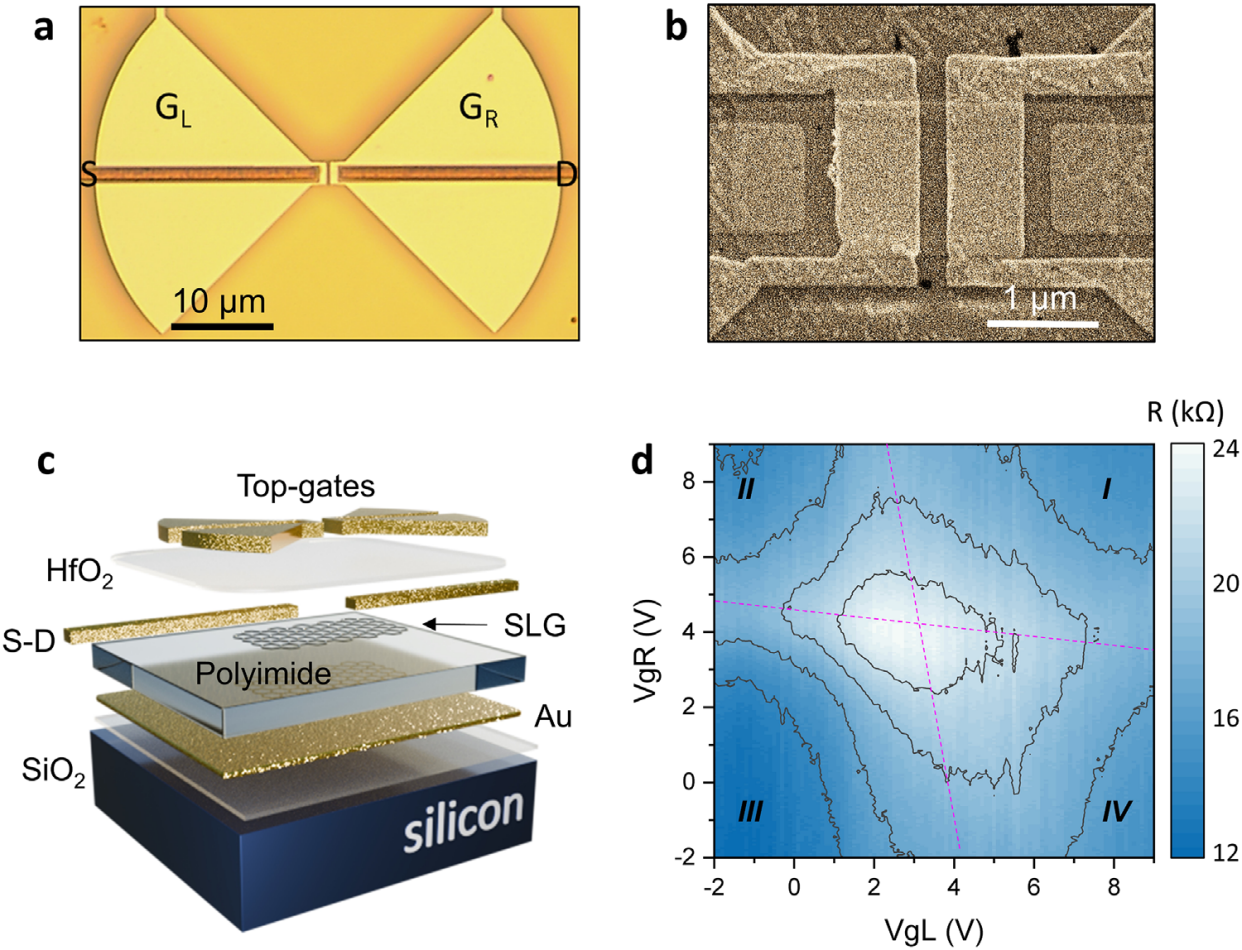
a) Optical microscope image of a prototypical device. The double‐split‐gate geometry enables the establishment of a *p‐n* junction at the center of the graphene channel, by applying oppositely polarized (with respect to the charge neutrality point) gate voltages on the left and right top‐gate electrodes. b) False‐color scanning electron microscope (SEM) image of the split‐gate GFET. c) Schematic 3D view of the fabricated device, with a display of all the different layers composing the photodetector structure. d) Electrical characterization of the dual‐gated graphene field‐effect transistor: resistance map as a function of the voltages applied to the left (*V*_gL_) and right (*V*_gR_) gate electrodes. A maximum resistance value of 24 kΩ is visible at the center of the map, corresponding to the simultaneous occurrence of the two CNPs, whose positions on the map are indicated by the two dashed magenta lines.

Importantly, the electric field at the center of the antenna is inversely proportional to the gap distance between the two antenna halves.^[^ ^52^ ^]^ Therefore, achieving a gap size ≈100 nm is of utmost importance to improve THz absorption and promote subsequent carrier heating in graphene. In order to maximize the spatial overlap between the THz field enhancement and the gradient in the Seebeck coefficient, we adopt a split‐gate geometry,^[^ ^30^ ^]^ comprising two top gates connected to the dipole antenna branches, that enable the spatial coincidence of Δ*T*_e_ and Δ*S*_b_ at the gap (**Figure** **2**a,b). With this strategy, smaller gap dimensions also correspond to a steeper electrostatically defined Δ*S*_b_.

A 3D view of the AgSS device, highlighting the different layers composing the structure, is provided in Figure 2c. To define the reflecting surface, we deposited a 200 nm thick layer of gold on a 300 µm thick silicon substrate, capped by 300 nm of thermal SiO_2_. We then spin‐coated the liquid‐phase polyimide spacer on the gold surface, targeting a thickness of 5 µm, and hardened the PI surface by baking at 300 °C. Details about this process are provided in the Experimental Section. Large‐area (≈1 × 1 cm^2^) CVD‐grown SLG is then transferred on the PI surface. The quality of the transferred material is then assessed by a combination of micro‐Raman spectroscopy and atomic force microscopy (AFM) experiments (see Supporting Information). The shape and size of the graphene channels are subsequently defined by a combination of aligned electron beam lithography (EBL) and reactive ion etching (RIE). EBL is further used to define the source (S) and drain (D) contacts, which are made of a thin film of Cr/Au (5/25 nm). The dielectric layer used for isolation between the top‐gates and the bottom layers is realized by atomic layer deposition (ALD) of a 30 nm thick layer of HfO_2_, grown at a temperature of 130 °C. This choice is motivated by the large dielectric constant (≈17) of HfO_2_, and by its relatively large electrical breakdown voltage (>300 MV m^−1^).^[^ ^53^ ^]^ The oxide layer simultaneously acts as a passivation layer for stable operation at room temperature and also provides isolation for electrostatic gating. Finally, the top‐gate electrodes (G_L_ and G_R_) are shaped as the two lobes of a bow‐tie antenna, with radius *r*_A_ = 24 µm and flare angle 90°. Split gate gaps <200 nm are obtained by fine‐tuning the EBL patterning parameters. The devices are then wire bonded to external circuitry (e.g., dual inline package). Further details about device fabrication are provided in the Methods section

### 2.2 Transport and optical measurements

The transport characteristics of the photodetectors are measured by monitoring the channel resistance (*R*) as a function of the voltages applied to the gate electrodes. To this end, we employed three *dc* source‐meters (Keithley, K2400), to independently control the source‐drain voltage (*V*_DS_), the left‐top‐gate voltage (*V*_gL_), and the right‐top‐gate voltage (*V*_gR_). Figure 2d displays the map of R as a function of both *V*_gL_ (horizontal axis) and *V*_gR_ (vertical axis) when *V*_DS_ = 1 mV. Both gates are swept over a voltage range between −2 and +9 V, and both show a CNP voltage (*V*_CNP_) at ≈+4 V, indicated by dashed magenta lines. These lines define four regions in the resistance map. Regions II and IV correspond to gate configurations where a *p‐n* junction is active at the center of the graphene channel. The *R(V*_G_) plot allows to estimate the electronic parameters of the graphene channel: field‐effect carrier mobility (µ_FE_), residual carrier density (*n*_0_), and contact resistance (*R*_c_). This is achieved using the fitting expression:^[^ ^54^ ^]^ *R*  =  *R*_c_ + (*L*_c_/*W*_c_)·(1/n_2d_eµ_FE_), where n_2d_  =  {n_0_^2^ + [C_g_/e·(V_g_−V_CNP_)]^2^}^−1/2^ is the gate‐voltage‐dependent carrier density, *L*_c_ is the length of the gated region, *W*_c_ is the channel width, and *C*_g_ = 0.5 µF cm^−2^ is the gate capacitance per unit area, determined by the gate oxide thickness and relative permittivity (ε_r_). For the *R* map shown in Figure 2d, we obtain the following parameters: µ_FE_ = 680 cm^2^V^−1^s^−1^, *n*_0_ = 5×10^12^ cm^−2^, and *R*_c_ = 13.3 kΩ for electrons, and µ_FE_ = 750 cm^2^V^−1^s^−1^, *n*_0_ = 3.5 × 10^12^ cm^−2^, and *R*_c_ = 11.5 kΩ for holes (results from the electrical characterization of other samples are reported in the Supporting Information). These values of mobility and residual carrier density are compatible with previous results obtained for CVD‐based graphene devices fabricated on Si/SiO_2_ substrates,^[^ ^19^ ^]^ indicating that the PI layer does not affect the SLG quality.

**Figure 3:**
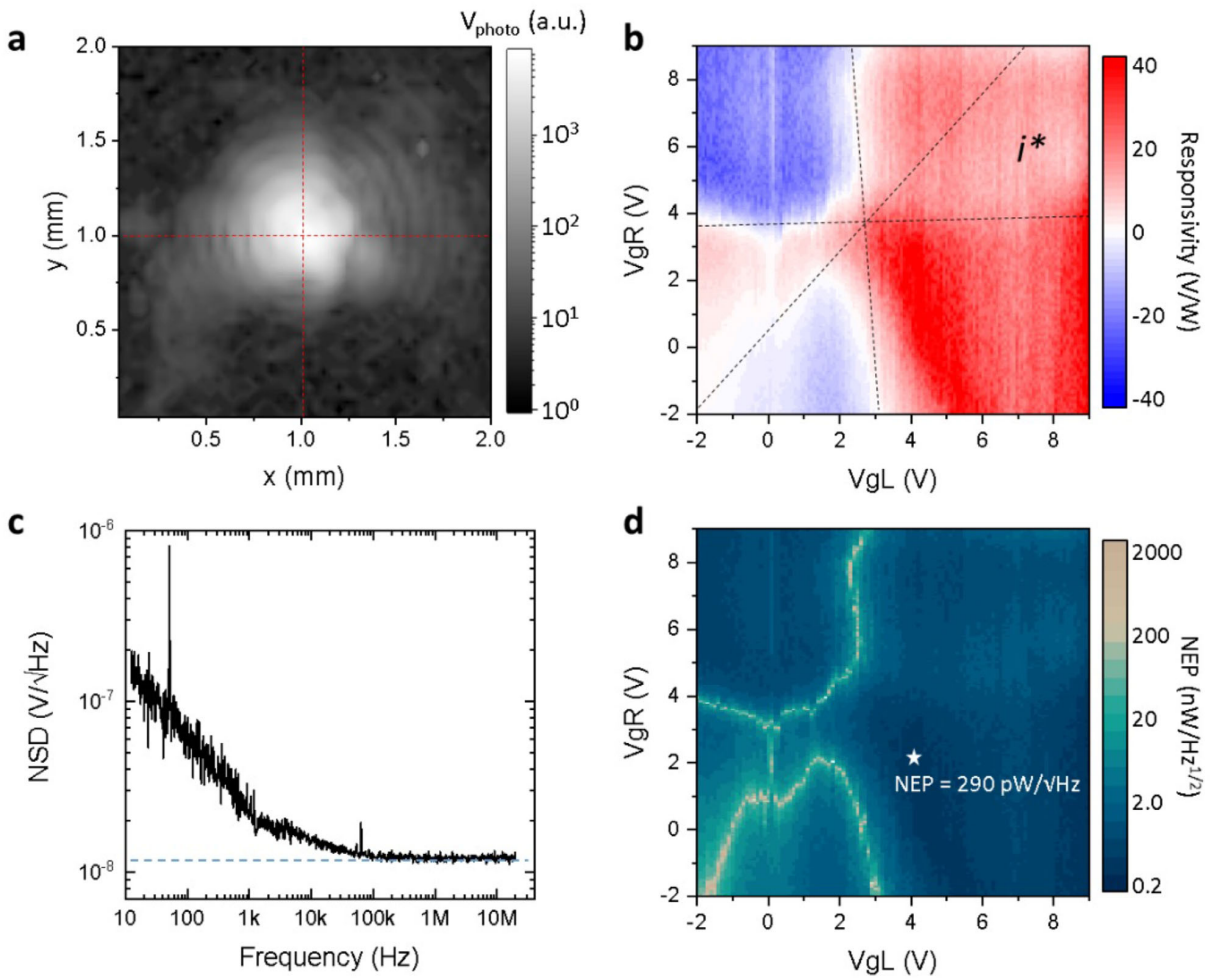
a) THz beam spot imaged by raster scanning the AgSS photodetector in the focal plane, over a 2 × 2 mm^2^ area. Logarithmic plot of the photovoltage, for an average impinging THz power of 400 µW. Eight concentric diffraction rings are visible outside the central spot. The bright circle at the center of the diffraction pattern, marked by the crossing point of the two dashed red lines, has FWHM 300 µm and 210 µm (approximated using a Gaussian fit) along the *x* and *y* directions, respectively. b) Gate‐dependent responsivity map at 2.86 THz, showing a six‐fold pattern typical of PTE response. Dashed lines are guides to the eye. c) Noise spectral density measured with a lock‐in frequency sweep, for *V*_gL_ = *V*_gR_ = 0 V. The dashed blue line is the theoretical thermal noise evaluated with the Johnson‐Nyquist formula. d) NEP map as a function of the two gate voltages, evaluated by approximating the detector noise figure with the thermal noise floor. Regions where the responsivity vanishes in b) correspond to regions where the NEP diverges in d). The gate voltage configuration, indicated by the white star, shows the condition where the minimum NEP value is achieved.

The detectors have been then investigated optically, by focusing the 2.86 THz light (wavelength λ = 105 µm) of a quantum cascade laser (QCL) on it. The QCL is cooled at ≈30 K in a Stirling cryostat, equipped with a high‐density polyethylene (HDPE) output window. The QCL is driven by a pulse wave with a repetition frequency of 40 kHz and a duty cycle of 4%, corresponding to an *on*‐state period of 1 µs. This pulse wave is further enveloped by a slower (1.333 kHz) square wave modulation, which is used as a reference for lock‐in small signal detection. Under this condition, a maximum ≈1 mW average output power is emitted. The QCL beam is focused by means of two THz‐transparent TPX (poly 4 methyl pentene‐1) lenses on the detector. The intensity distribution in the focal plane has been acquired with our AgSS detector: the device output photovoltage (Δu) is collected at the D electrode while keeping S grounded. Δu is then pre‐amplified and recorded with a lock‐in. The AgSS is raster‐scanned over the focal plane (*xy* in **Figure** **3**a) by means of high‐precision motorized stages. The beam diffraction pattern (Airy disk), displayed in Figure 3a shows >8 orders of diffraction, and a signal‐to‐noise ratio (SNR) exceeding 2000. Such a result reflects the highly sensitive performance of our detector, as there is a very small fraction of total incident beam power (*P*_t_ = 560 µW in Figure 3a) distributed among the diffraction rings.^[^ ^55^ ^]^ By approximating the beam profile with an asymmetric 2D Gaussian function,^[^ ^29^ ^]^ we retrieve a full width at half maximum (FWHM) of 300 µm along the horizontal (*x*) axis and 210 µm along the vertical (*y*) axis, which correspond to a beam spot area S_spot_ = 5 × 10^4^ µm^2^.

The detector responsivity (*R*_v_) is then retrieved from the photovoltage (*V*_photo_) measured by the lock‐in, after normalizing it to the diffraction limited area (*S*_λ_).^[^ ^56^ ^]^ In this approximation, the power coupled to the photodetector (*P*_a_) can be calculated as *P*_a_ = *P*_t_×(*S*_λ_/*S*_spot_), where P_t_ is the total input power at the detector position, and *S*_λ_ = λ^2^/4 = 2750 µm^2^ is the diffraction limited area. We note that the area normalization takes into account the fact that, when the device's effective area is much smaller than the beam spot size, only a fraction of the total power (*P*_t_) is actually coupled to the photodetector. Different normalization strategies have been discussed in previous works;^[^ ^29^, ^56^, ^57^ ^]^ the diffraction limit normalization gives a reasonable estimate of the maximum response that can be obtained considering the detector together with an optimized focusing system (e.g., using a hemispherical lens) and is widely used in literature for comparing the performances among different photodetectors.^[^ ^19^, ^20^, ^21^, ^29^, ^30^, ^39^, ^40^, ^56^, ^57^ ^]^ *R*_v_ is then calculated from the expression:^[^ ^20^ ^]^

(1) $$R_{v}=\left(\frac{2\pi \sqrt{2}}{4\gamma }\cdot V_{\mathrm{photo}}\right)\cdot \frac{1}{P_{t}}\left(\frac{S_{\mathrm{spot}}}{S_{\lambda }}\right)$$

where the first term between round brackets is the detector photovoltage Δu, and γ = 1000 is the gain of the voltage preamplifier (DL Instruments, model 1201, bandwidth 100 kHz). Since the AgSS detector operates through the PTE detection mechanism, its *R*_v_ is expected to be strongly influenced by the voltages applied to the gate electrodes, i.e., by the presence of a Seebeck coefficient gradient at the center of the graphene channel. Specifically, the PTE photovoltage Δu_PTE_ is proportional to Δ*S*_b_ on the left‐ and right sides of the junction: Δu_PTE_ = Δ*T*_e_ × (*S*_L_ − *S*_R_), where Δ*T*_e_ is the THz‐induced electronic temperature increase at the junction. To verify the presence of PTE rectification, we then measure *R*_v_ as a function of *V*_gL_ and *V*_gR_. The results of this experiment are shown in Figure 3b.

The *six‐fold* pattern, observed in the measured photovoltage, is attributed to the non‐monotonic gate voltage dependence of S_L_ and S_R_ on each side of the junction, which leads to multiple sign changes of Δ*S*_b_. We note that other photodetection mechanisms, such as the overdamped plasma wave,^[^ ^20^ ^]^ or the resonant plasmon‐assisted effects,^[^ ^58^ ^]^ may also contribute to the THz photoresponse in a GFET, at room temperature, under zero‐bias operation. However, the emergence of a six‐fold pattern in the responsivity map of a split‐gate p‐n junction is a unique fingerprint of a dominant hot‐carrier‐assisted PTE effect in SLG.^[^ ^37^ ^]^ Additionally, we note that for *V*_gL_ values > CNP (right‐half of the *R*_v_ map), the device photoresponse does not show a full transition to negative values, in the region marked with *i* ^*^. We ascribe this to a weak inhomogeneity between the left and right portions of the channel, which prevents Δ*S*_b_ from changing signs in the investigated gate‐voltage range. Interestingly, the device *R*_v_ reaches values >40 V/W when the *p‐n* junction is active. This value is ≈1 order of magnitude larger than previously reported results on detectors based on CVD‐grown graphene, operating around 3 THz^[^ ^19^, ^40^ ^]^ and in line with the performance reported for high‐quality hBN‐encapsulated devices.^[^ ^29^, ^39^ ^]^

Additionally, we characterized the NEP of our photodetectors. This is done following two parallel methods. The first is the direct measurement of the detector noise spectral density (NSD). This is done using a high‐frequency lock‐in amplifier and measuring the root means square (RMS) of the noise amplitude while sweeping the internal oscillator frequency from 10 Hz to 20 MHz^[^ ^59^ ^]^ (Figure 3c). The noise figure shows a 1/*f* contribution to the noise up to a frequency of ≈10 kHz, above which the noise level flattens to the thermal (Johnson‐Nyquist) background: N_th_ = (4k_B_RT)^1/2^, where k_B_ is the Boltzmann constant, R is the resistance of the SLG channel, and *T* is the device temperature (295 K). We observe that the ratio between the NSD measured at the lock‐in modulation frequency (1.333 kHz) and the high‐frequency asymptote (N_th_) is just a factor of 1.5. This small discrepancy, combined with the typical invariance of *R*_v_ as a function of envelope modulation frequency,^[^ ^19^ ^]^ justifies the use of a second strategy to evaluate the detector's NEP, which consists of approximating the NSD with the thermal noise figure. This allows us to use the maps of *R* and *R*_v_ as a function of the gate voltages to calculate the NEP(*V*_gL_, *V*_gR_) map shown in Figure 3d as NEP = (4k_B_ *RT*)^1/2^/*R*_v_. By using this approximation, we obtain a minimum NEP < 300 pWHz^1/2^.

Finally, in order to provide direct evidence of the role played by the polyimide spacer, we compare the figures of merit obtained with the optimized PI thickness (5 µm) with those measured for a PI thickness corresponding to the quarter‐wavelength distance (≈15.5 µm). The optical responsivity for the latter device is ≈1 V W^−1^, corresponding to minimum NEP values of ≈20 nWHz^−1/2^. The observed difference in responsivity between the two geometries is comparable with the difference between the simulated in‐plane field enhancement (Figure 1), which predicts an *R*_v_ ratio ≈20. The characterization of a quarter‐wavelength distance is reported in the Supporting Information file.

**Figure 4:**
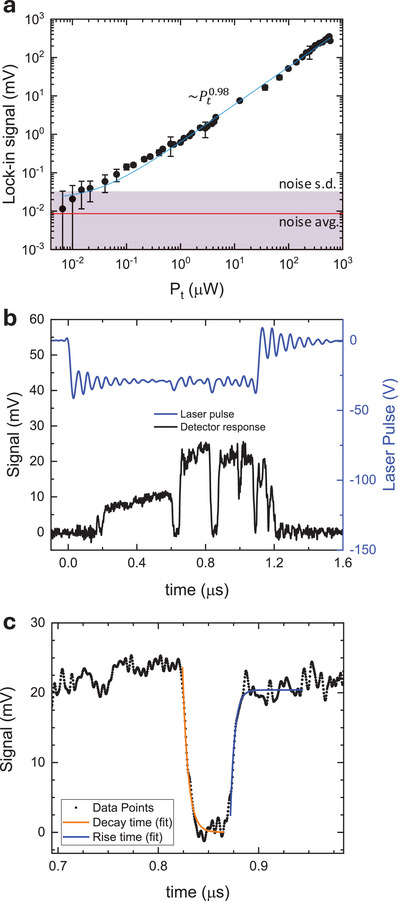
a) Dynamic range characterization: lock‐in signal as a function of impinging optical power, showing a linearity range >4 orders of magnitude. The blue line is a power fit to the experimental data. The red line is the instrumental (laboratory) noise floor. The red‐shaded area represents the standard deviation of the noise floor, measured with a lock‐in integration time of 300 ms. b,c) Response time evaluation. Time traces of the photovoltage under pulsed illumination were recorded with a fast oscilloscope. A response time ≈4 ns is estimated, corresponding to a bandwidth of ≈30 MHz.

We finally tested the power dynamic range and the speed of the receiver. **Figure** **4**a represents the measurement of the response of our photodetector (*V*_photo_) as a function of the optical power in the beam spot area (*P*_t_), from which we can evaluate its dynamic range, i.e., the power interval within which the detector is capable to provide a measurable response whose magnitude is linearly proportional to the input optical intensity. The optical power at the focal point is controlled through the voltage applied to the QCL and calibrated using pyroelectric and THz thermopile sensors as primary standards. Although our device measures just a fraction of P_t_, it exhibits a sensitivity beyond the lower limit of the pyroelectric sensor. Thus, to study the minimum detectable power (MDP), we applied a set of calibrated THz attenuators made of metalized wedged silicon wafers that enable to explore the full range of detector sensitivity over more than 4 orders of magnitude. We found an MDP level below 10 nW. Fit of the experimental data with a simple power law *V*_photo_ ≈ *P*_t_^γ^ (dashed line in Figure 4a) confirms that the detector fulfills conditions of linearity (γ = 0.98) across almost the entire considered range of powers, with a saturation intensity *I*_sat_ = 1.1 W cm^−2^. This behavior can be explained by the so‐called weak‐heating regime:^[^ ^60^ ^]^ as long as the THz intensity is smaller than *I*_sat_, the THz‐induced Δ*T*_e_ at the junction position is a fraction of the background (room) temperature.

To evaluate the response time, we characterized the thermal instabilities of the QCL beyond its negative differential resistance (NDR) threshold, corresponding, in the present case, to a voltage pulse <−30 V. In this regime, an intermittent output power with characteristic time constants of ≈1 ns is present. This intermittent state can be recorded by a sufficiently fast photodetector. We then measure time‐resolved photodetection waveforms from our AgSS receiver, illuminated by the pulsed QCL, with a fast oscilloscope (Tektronix, MSO64B 6‐BW‐4000, 4 GHz bandwidth). In order to enhance the bandwidth of the detection system, we replace the low‐noise voltage preamplifier, employed for the lock‐in measurements, with a faster transimpedance amplifier having a bandwidth of 80 MHz and gain of 10^4^ V A^−1^ (Femto, DHPCA). Figure 4b shows two waveforms. The upper one (blue line) is the QCL voltage pulse, with an on‐state period of 1 µs. The lower one (black line) is the photodetector signal time trace, which presents a sequence of transitions between *on* and *off* states, as a consequence of light intermittence. By zooming on one of the transitions (Figure 4c), and fitting the signal waveform with exponential functions,^[^ ^29^ ^]^ we obtain the detector's rise‐time *τ*_ON_ = 4.2 ± 0.1 ns, and fall‐time *τ*_OFF_ = 5.5 ± 0.1 ns, corresponding to a bandwidth BW = 30 MHz. We compare this result with the pulse characterization performed with a fast (200 MHz) cryogenically cooled superconducting bolometer (Scontel), which we use as a primary standard for QCL rise‐time characterization, and for which we obtain *τ*_ON_ = 3.7 ± 0.1 ns, and *τ*_OFF_ = 5.2 ± 0.2 ns (see Supporting Information). This demonstrates that the 30 MHz bandwidth measured for our AgSS photodetector is setup‐limited by the rise and fall times of the intermittent THz radiation emitted by the QCL, when it is driven into the instable NDR regime.

## 3 Conclusion

Compared to other receiver systems, in which the CVD‐SLG *p‐n* junction is coupled to planar antennas on a semiconductor substrate, the AgSS scheme achieves an order of magnitude improvement in the recorded responsivity with a consequent NEP reduction. This improvement is attributed to the optimized SLG‐radiation coupling. Notably, this is accompanied by a huge power dynamic range (>4 orders of magnitude), which can enable the usability of AgSS receivers in THz communication schemes.^[^^61]^ The device scalability and the possibility to transfer the same concept to alternative substrates prospects major impacts in flexible electronics, maintaining the advantages of low size, weight, and power consumption (SWaP) provided by SLG‐based PTE detection.^[^ ^18^ ^]^ Furthermore, the same device concept can be extended to other frequency ranges, benefitting from an expanded parameter space for performance optimization, compared to the more traditional antenna‐on‐substrate schemes. Ultimately, the careful selection of materials could potentially allow for operation within wearable platforms,^[^ ^46^ ^]^ given the biocompatibility of the materials used.

## 4 Experimental Section

### Deposition of the Polyimide Spacer

PI2574 (HD Microsystems) was used as a liquid polyimide precursor. PI2574 was spin‐coated onto the Au/SiO₂/Si substrate. The spin‐coating was performed at different speeds (4300, 5100, and 7700 rpm) for 30 s, following the technical specifications in the PI2574 datasheet. A film thickness of 5 µm was achieved at 4300 rpm. However, optical profilometry revealed that while the central region of the film is uniform, the outer region exhibits non‐uniformity. An edge bead removal (EBR) technique was then employed to enhance film uniformity at the edges. EBR was conducted at 6000 rpm for 5 s at the end of the main rotation step, to remove excess precursor from the sample edges. Finally, the film was cured on a hotplate at 300 °C for 60 min to harden the surface and complete the process.

### Device Fabrication

The devices presented in the present work were fabricated on a (300 µm) Si substrate covered by a thin (300 nm) SiO_2_ layer. The reflector layer of 200 nm thick Au with a 5 nm buffer layer of Cr was grown on the substrate via thermal evaporation technique. The gold was subsequently coated by the polymer spacer polyimide (PI) 2574. A set of samples with various thicknesses of polymer was prepared by fine‐tuning the spin‐coating and baking processes.

The initial stage of the fabrication was the wet transfer of an SLG onto the polyimide surface. The single‐layer graphene (SLG) grown via chemical vapor deposition (CVD) technique was utilized. The patterning of the device structure was performed using electron beam lithography (EBL). First, negative tone photoresist ma‐N 2403 was employed to mask the rectangularly (4 to 1.6 µm) patterned SLG channels. This resist was developed using aqueous‐alkaline tetramethylammonium hydroxide (TMAH) based developer ma‐D 525. The residual graphene was removed by means of reactive ion etching using oxygen plasma. EBL patterning of the metallic contacts (source and drain terminals, S and D respectively), insulating layers, and the antennas (left and right gate terminals, G_L_ and G_R_ respectively) was done with the polymethyl methacrylate (PMMA) resist AR‐P 679.04. In these processes, we applied methyl isobutyl ketone (MIBK) based developer AR 600–56. First, the Cr/Au (5/25 nm) source and drain contacts were deposited using the evaporation technique. Subsequently, a thin (≈30 nm) insulating layer of HfO_2_ was grown at 130 °C via atomic layer deposition (ALD) in order to prevent current flow between the channel and the antenna electrodes. This layer enables electrostatic gating and, in addition, plays the role of passivation layer for stable ambient operation. Finally, the bow‐tie antenna with radius r = 24 µm and flare angle 90° was patterned and metalized (Cr/Au 5/100 nm).

## Conflict of Interest

The authors declare no conflict of interest.

## Supporting information



Supporting Information

## Data Availability

The data that support the findings of this study are available from the corresponding author upon reasonable request.
