# Supplementary material for: Efficient Large‐Area Graphene p‐n Junction Terahertz Receivers on an Integrated Optical Platform
Source: Small Methods. 2025 Apr 17;9(11):2500083. doi: 10.1002/smtd.202500083 (PMC12641380; doi:10.1002/smtd.202500083)
Supplement: Supplementary file 1 — Supporting Information [file SMTD-9-2500083-s001.docx]

**Supporting Information for**

**Efficient large-area graphene *p-n* junction terahertz receivers on an integrated optical platform**

**Leonardo Viti^1^, Vladimir Pushkarev^1^, Syed Muhammad Abouzar Sarfraz^2^, Gaetano Scamarcio^2^, Miriam S. Vitiello^1*^**

*^1^NEST, CNR-Istituto Nanoscienze and Scuola Normale Superiore, Piazza San Silvestro 12, Pisa 56127, Italy*

*^2^Dipartimento Interateneo di Fisica, Università degli studi di Bari, Via Amendola 173, Italy*

**Table of Contents**

- S1- Atomic force microscopy Pag. 2
- S2- Raman spectroscopy Pag. 2
- S3- Characterization of additional devices Pag. 3
- S4- Comparison between expected and measured responsivity Pag. 5
- S5- Fabrication Challenges Pag. 6
- S6- Response time measurement Pag. 8

**S1- Atomic Force Microscopy**

We characterize the topography of the SLG surface after transfer on top of polyimide (PI) by atomic force microscopy (AFM). **Figure S1** shows an AFM image of a 50×50 μm^2^ area.

**
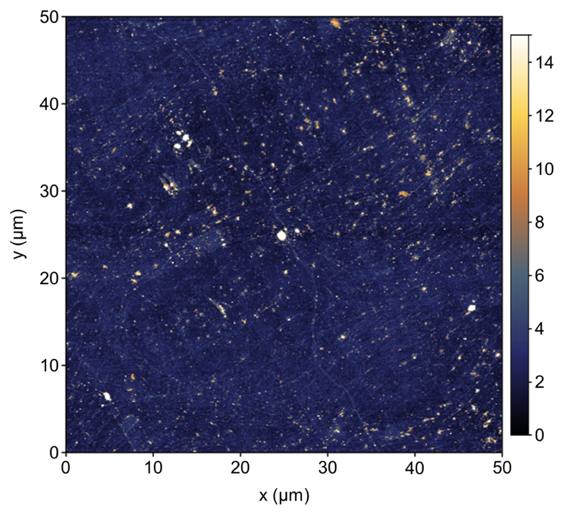
**

**Figure S1.** Atomic force microscopy image of the surface of SLG transferred on polyimide. From the analysis of the topography, we infer a root means square roughness of 2.39 nm and a mean roughness of 0.98 nm.

**S2- Raman spectroscopy**

Raman spectra are acquired on SLG, transferred onto polyimide, by using a Horiba spectrometer equipped with an 1800 gr mm^−1^ grating, ×100 objective lens with a numerical aperture of 0.95, producing a spot size of ≈1 µm. All spectra are recorded under ambient conditions at a wavelength of 638 nm, with an incident laser power lower than 5 mW to avoid laser heating.

**Figure S2a** shows typical Raman spectra of PI and SLG on PI. The Raman response of PI presents multiple peaks at frequencies < 2000 cm^-1^, attributed to molecular vibrations. However, the characteristic G and 2D peaks of graphene, located at ≈1580 cm^-1^ and ≈2650 cm^-1^, respectively, are clearly identifiable. We perform a statistical analysis acquiring Raman spectra at different positions on SLG after transfer onto PI. The results of the analysis, which compares the 2D-peak frequency to the G-peak frequency across various positions on the sample, are presented in **Figure S2b**. The data indicate the presence of tensile strain and a significant doping level, with Fermi energy, E_F_, exceeding 350 meV.

**
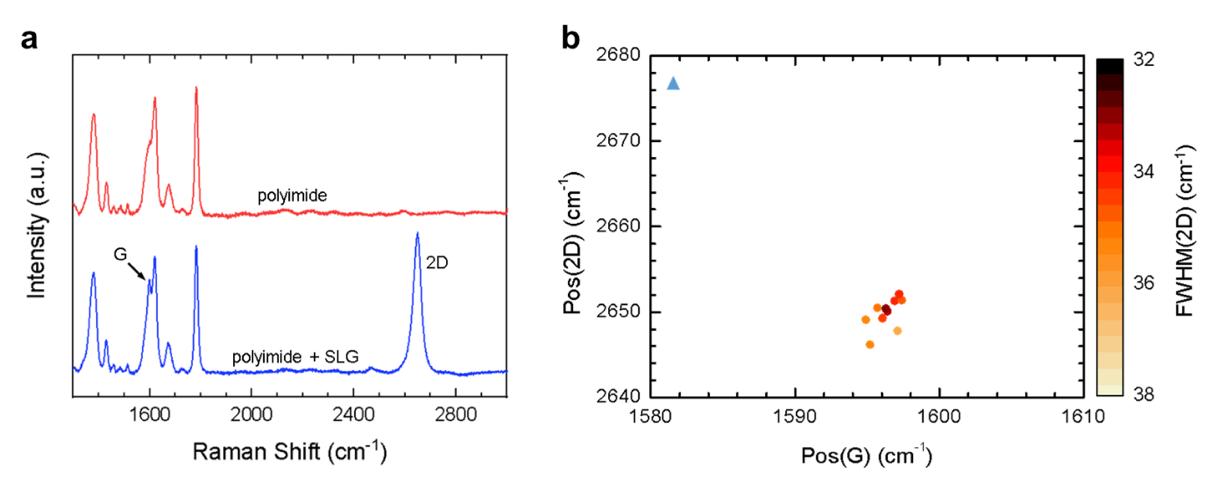
**

**Figure S2.**  **(a)** Raman spectra of polyimide (red line) and SLG transferred onto polyimide (blue curve). **(b)** 2D-peak vs G-peak frequency extracted from Raman spectra acquired at different positions on the sample. The color bar represents the values of full width at half maximum of the 2D-peak. The blue triangle marks the position that SLG, not affected by strain or doping, should occupy on the map. The dispersion of the data points suggests the presence of tensile strain.

**S3- Characterization of additional devices**

We fabricated and characterized different batches of AgSS photodetectors, adjusting the thickness (*h*) of the PI spacer to assess its influence on the main figures of merit.

In the first run, we devise AgSS with *h* set at the standard Salisbury mirror distance (quarter-wavelength). For a radiation frequency of 2.86 THz, the effective wavelength in the spacer layer is λ_PI_ = 56 μm, given the polyimide refractive index *n*_PI_ = 1.85. We then fabricate a batch of samples using *h* = λ_PI_/4 ≈ 15 μm. This allows us to clearly understand the role of the spacer layer and the importance of its thickness. **Figure S3** displays the electrical and optical characterization of an AgSS with *h* = 15 μm. The reported electrical resistance and responsivity curves as a function of gate voltage (left-gate-voltage sweep, while keeping V_gR_ = 0 V) show that for electrical performance in-par with the optimized AgSS, the maximum voltage responsivity is ~40 times lower with respect to devices realized with *h* = 5 μm, demonstrating the accuracy of the electromagnetic simulations.


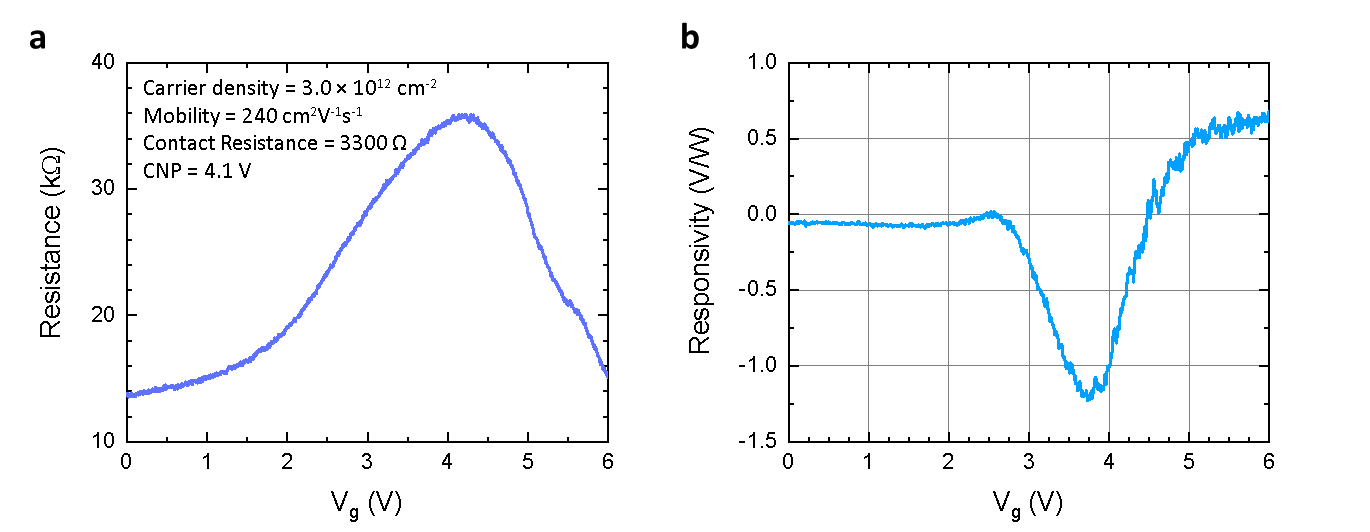


**Figure S3.** Electrical (a) and optical (b) characterization of an AgSS device with *h* = 15 μm. Electrical parameters retrieved from the fit of the resistance curve are displayed. The double sign change in the responsivity curve is a signature of a PTE driven photoresponse.

To further characterize the influence of the spacer thickness (parameter *h*) on the photodetector response and the reproducibility of the obtained results, we fabricated additional AgSSs with *h* = 4.8 μm. The results of the optical characterization are displayed in **Figure S4**. The three devices show similar responsivity (R_v_) curves as a function of the voltage applied on the right gate (V_gR_), while V_gL_ = 0 V. The sign of R_v_ changes twice, which is indicative of a photo-thermoelectric (PTE) contribution, with a maximum observed at large positive values of V_gR_, *i.e.* when the *p-n* junction is activated. Remarkably, the maximum responsivity is between 30 VW^-1^ and 50 VW^-1^ for the three detectors, and their charge neutrality points (CNPs) occur for V_gR_ values between 1 V and 2 V.


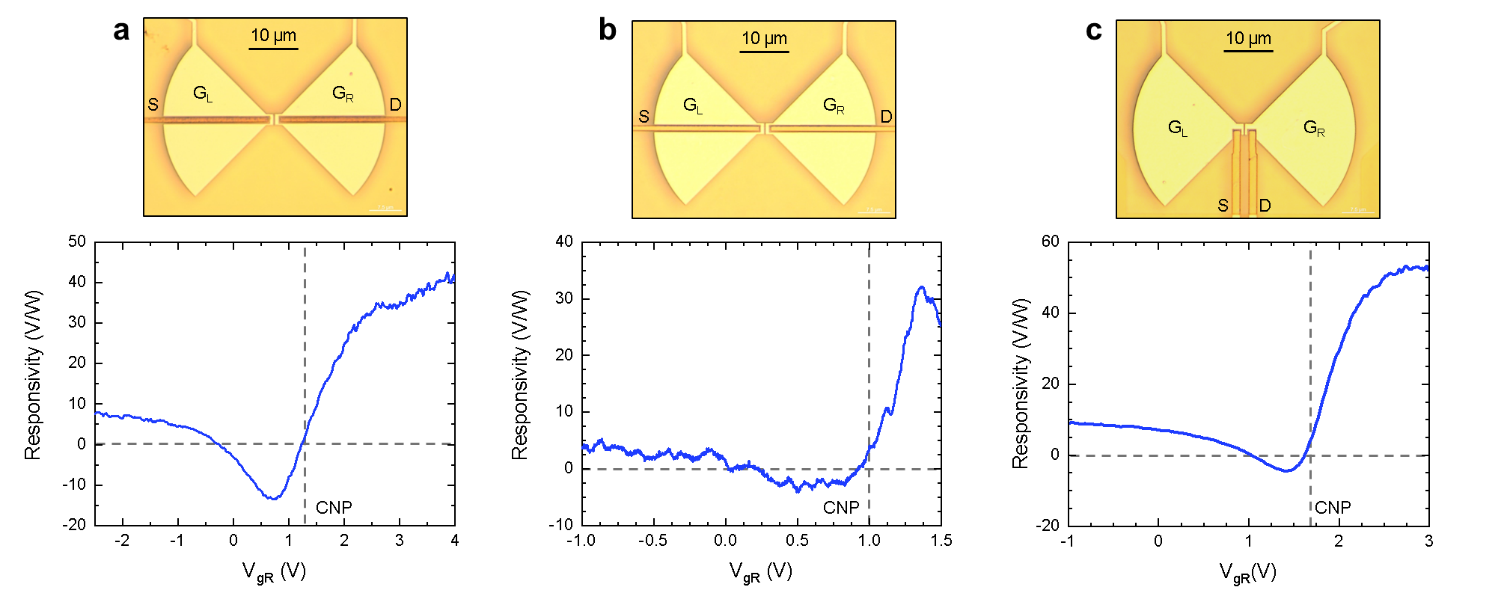


**Figure S4.** (a-c) Optical microscope images and responsivity curves as a function of V_gR_ measured for three AgSS devices fabricated with *h* = 4.8 μm. The device in (c) features a slightly different antenna geometry: bow-tie with source and drain lines exiting parallel from a side of the graphene channel. The double sign change in the responsivity curves is a signature of a PTE driven photoresponse.

**S4- Comparison between expected and measured responsivity**

This section presents a qualitative comparison between the simulated and measured photoresponse as a function of the thickness (*h*) of the PI spacer. As illustrated in **Figure 1c** (main text), the design parameter *h* is expected to significantly impact the field-enhancement at the center of the planar antenna, *i.e.* on the SLG channel. In SLG, the increase in electronic temperature (ΔT_e_) due to intraband transitions (free-carrier, or Drude, absorption) is proportional to the square of the in-plane component of the electric field (E_x_)^2^. Consequently, the quantity |E_x_|^2^ is proportional to the thermoelectric photovoltage, Δu_PTE_, expressed by the relation Δu_PTE_ = ΔT_e_ × Δ*S*_b_, where Δ*S*_b_ = *S*_b_(left) – *S*_b_(right) represents the gradient of the Seebeck coefficient across the junction, *i.e.* at the center of the antenna. As a result, the responsivity of AgSS detectors as a function of *h* is expected to align with the simulated trend of |E_x_|^2^(*h*). This comparison is displayed in **Figure S5**, which shows a good qualitative agreement between the simulated and measured responsivities. We note that a quantitative analysis of the device’s internal responsivity requires a precise knowledge of many device parameters, including antenna efficiency, SLG THz conductivity as a function of Fermi level, carrier scattering and relaxation mechanisms,^1^ and the Seebeck coefficient.^2^ The evaluation of these parameters typically relies on reasonable approximations (e.g. using the Mott equation or the Boltzmann formalism to calculate *S*_b_),^2^ but their cumulative effect may lead to significant inaccuracies, rendering quantitative comparisons somewhat speculative.

**Figure S5.** Comparison between simulated in-plane electric field enhancement (E_x_^2^) and measured detector responsivity as a function of spacer thickness (*h*).

**S5- Fabrication challenges**

The fabrication of nano-devices on polymeric substrates presents challenges that do not impact processes performed on solid-state substrates. In the following, we outline the main issues faced, along with a potential roadmap for developing graphene-based THz photodetectors on flexible substrates.

*Charging effects*. Plastic substrates such as PET, Melinex, or Kapton are electrical insulators. This characteristic complicates the use of high-current, high-resolution electron beam lithography (EBL), especially when the polymer thickness exceeds 2-3 μm. Additionally, this feature makes nano-devices more susceptible to damage from discharge or unbalanced potentials. This problem can be mitigated by using a thin layer of a conductive protective coating (AR-PC 5090, AllResist) during EBL exposures.

*Graphene visibility*. The optical contrast of single-layer graphene (SLG) on polyimide is quite poor, even under red-channel illumination. This hampers the easy identification of wrinkled or defective areas in transferred large-area SLG films. This issue can be partially alleviated by using microscopes equipped with dark-field illumination.

*Thermal damage*. Plastic materials are prone to thermal damage, and the nano-fabrication procedures must ensure their mechanical stability. This requires extra care and gentle temperature ramps when baking samples or during deposition steps requiring elevated temperatures, such as atomic layer deposition (ALD) and thermal evaporation. In particular, typical thermal ALD temperatures of ~ 300°C are unsuitable and low-temperature processes should ideally be employed (e.g., we deposited HfO2 at 130°C).^3^

*Wire Bonding*. Ultrasonic wire bonding on polyimide can be critical because of heating-induced polymer softening: ultrasonic energy heats the PI beneath the metallic bonding pads (**Figure S5**), reducing the material’s stiffness. Consequently, the softened polymer absorbs ultrasonic energy, leading to a failure in bond formation between the metallic (Al) wire and the pads, thereby reducing the wire bonding yield.^4^ To address this issue, we have strengthened the bonding pads with a further evaporation of Ti/Au (20/100 nm) at the end of the fabrication procedure. This ensures robustness to the metallic pads, improving the yield. It is important to note that electrical insulation is ensured by defining the two gate electrodes over the 30 nm insulating layer of HfO_2_, which extends beneath the bonding pads.


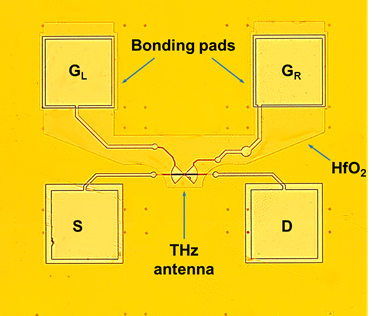


**Figure S6.** Optical image of the full device, showing the pads used for wire bonding to the external circuitry.

*Roadmap towards flexible electronics*. Previous works on graphene-based THz photodetectors on flexible substrates relied on fabricating graphene field-effect transistors (GFETs) directly on the substrate.^5^ While this approach is simpler from a nano-fabrication perspective, it requires modifications to the photodetector design based on the specific flexible material employed as a substrate, e.g. on its refractive index. In contrast, the proposed AgSS approach allows for substrate-independent fabrication, as the optical coupling is determined solely by the design above the gold reflector.

A potential challenge in fabricating nano-structures on flexible plastic substrates is connected to bowing and deformation resulting from excessive heating. Such deformations can adversely affect lithography steps that require a precise adjustment of the focal depth of the patterning beam, whether it be an electron beam or a photon beam. One possible strategy to circumvent this issue relies in the use of transfer printing techniques.^6^ These methods enable the fabrication process of the spacer and GFET detector on a rigid platform, followed by transfer-printing them onto a flexible substrate at the end of the fabrication process. We envision that transfer printing can facilitate the transition from laboratory-based fabrication to large-scale production of graphene-based flexible devices.

**S6- Response time measurement**

The emission of the quantum cascade laser (QCL), biased in the negative differential resistance (NDR) regime, is characterized by an intermittent emission with expected time constants ~1 ns. To characterize this emission state, we employed a commercial fast photodetector, a cryogenically cooled superconducting bolometer (Scontel), with a bandwidth > 200 MHz, therefore capable of capture rise and fall times shorter than 0.8 ns. **Figure S7** shows the time trace obtained by the bolometer when the QCL is driven in the NDR region. The light pulse detected by the bolometer has a different shape compared to the electrical pulse, because of the intermittent emission and of the small time-delay induced by the length of the cables employed to drive the QCL and read out the detector’s signal. Similar distortions of the light pulse are observed with the AgSS photodetector.

By applying the same fitting functions to quantify the response time of the bolometer, we obtain *τ*_ON_ = 3.7 ± 0.1 ns, and *τ*_OFF_ = 5.2 ± 0.2 ns. These time constants are comparable with the ones obtained by the AgSS, and significantly slower than the speed of the bolometer. Therefore, our experimental setup is intrinsically limited by the speed at which the QCL turns *on* and *off* during its intermittent emission.

**Figure S7.** Characterization of the intermittent emission of the THz QCL in the NDR regime. Black trace (left vertical axis): time waveform recorded by a fast (200 MHz) cryogenically cooled bolometer. Rise and fall times are calculated from exponential fits to the data. Blue trace (right vertical axis): time waveform of the electrical voltage pulse applied to the QCL.

**References**

[1] M. Mittendorff, S. Winnerl, T. E. Murphy, *Adv. Optical Mater.* **2021**, 9, 2001500. <https://doi.org/10.1002/adom.202001500>

[2] M. Asgari, L. Viti, O. Balci, S. M. Shinde, J. Zhang, H. Ramezani, S. Sharma, A. Meersha, G. Menichetti, C. McAleese, B. Conran, X. Wang, A. Tomadin, A. C. Ferrari, M. S. Vitiello, *Appl. Phys. Lett.* **2022**, 121, 031103. <https://doi.org/10.1063/5.0097726>

[3] A. Paghi, S. Battisti, S. Tortorella, G. De Simoni, *J. Appl. Phys.* **2025**, 137, 044103. <https://doi.org/10.1063/5.0250428>

[4] N. B. Jaafar, R. Damalerio, IEEE 19th Electronics Packaging Technology Conference (EPTC), Singapore **2017**, 1. <https://doi.org/10.1109/EPTC.2017.8277435>.

[5] X. Yang, A. Vorobiev, A. Generalov, M. A. Andersson, J. Stake, *Appl. Phys. Lett.* **2017**, 111 (2), 021102. <https://doi.org/10.1063/1.4993434>

[6] A. Carlson, A. M. Bowen, Y. Huang, R. G. Nuzzo, J. A. Rogers, *Adv. Mater.* **2012**, 24, 5284. <https://doi.org/10.1002/adma.201201386>
